# Supplementary figures and images for: Overall Survival of Hungarian Cancer Patients Diagnosed Between 2011 and 2019, Based on the Health Insurance Fund Database
Source: Cancers (Basel). 2025 May 15;17(10):1670. doi: 10.3390/cancers17101670 (PMC12110003; doi:10.3390/cancers17101670)

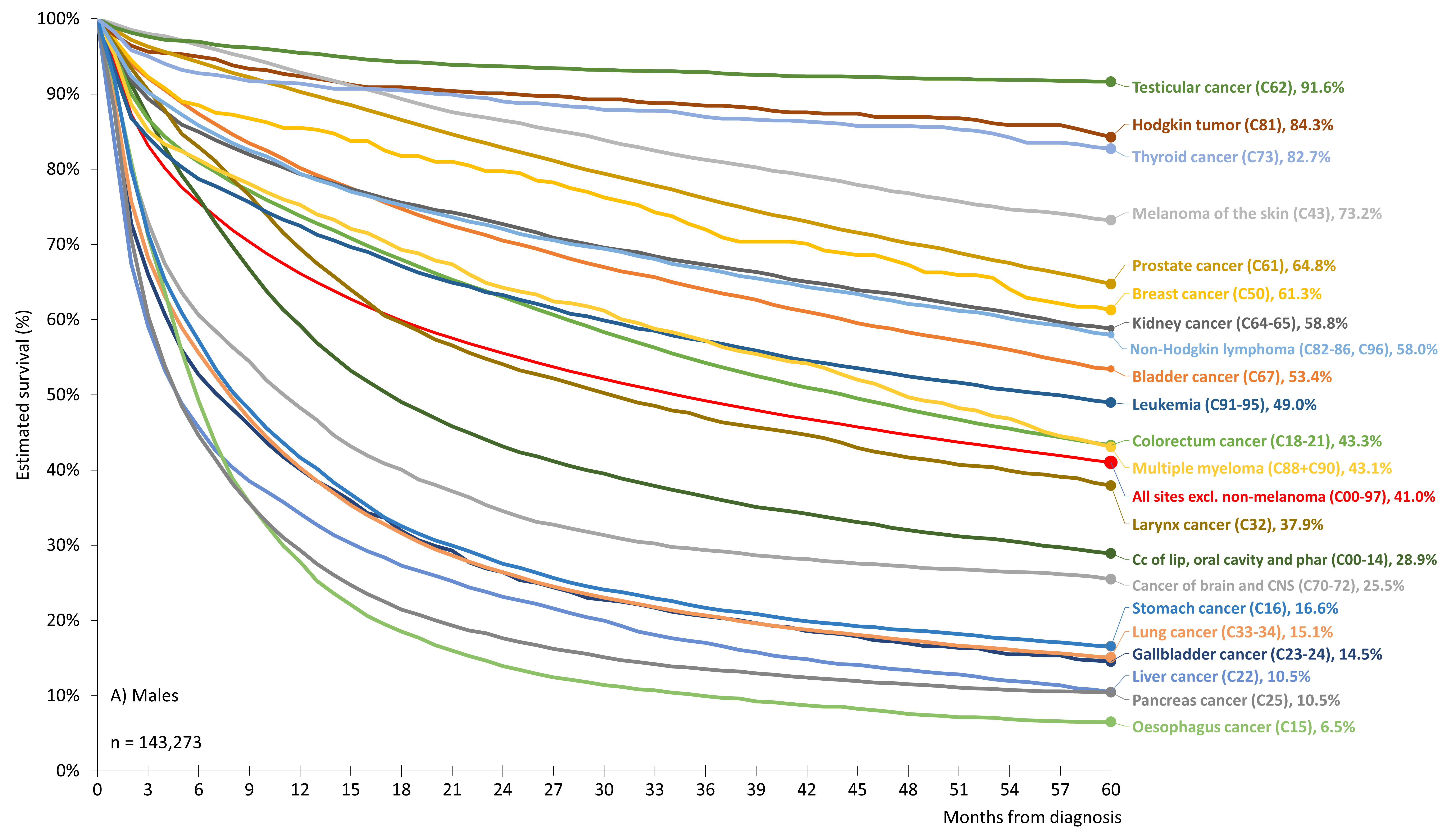

Supplement: Supplementary file 1 [file cancers-17-01670-s001.zip › Supplementary Figure S1A.png]

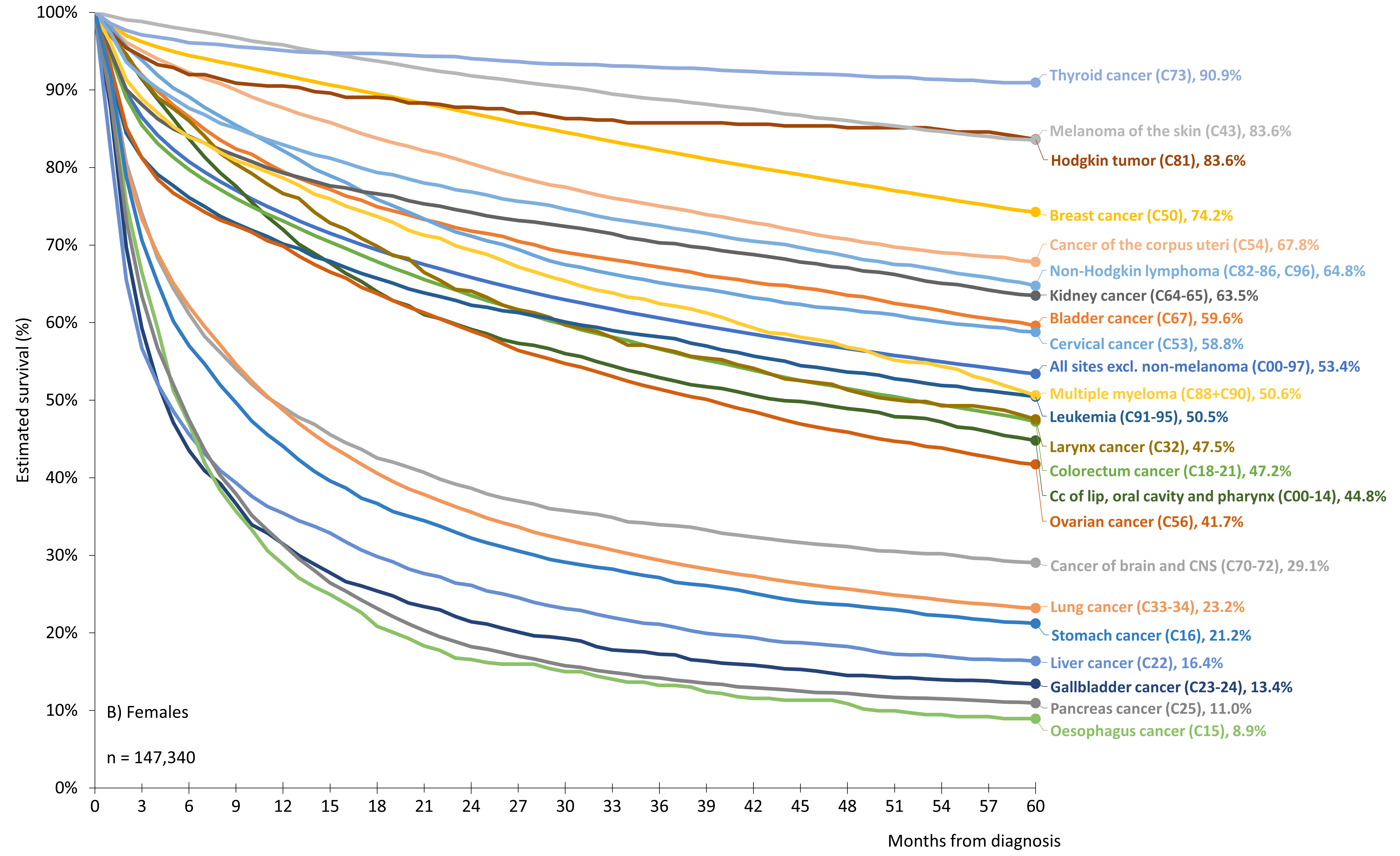

Supplement: Supplementary file 1 [file cancers-17-01670-s001.zip › Supplementary Figure S1B.png]

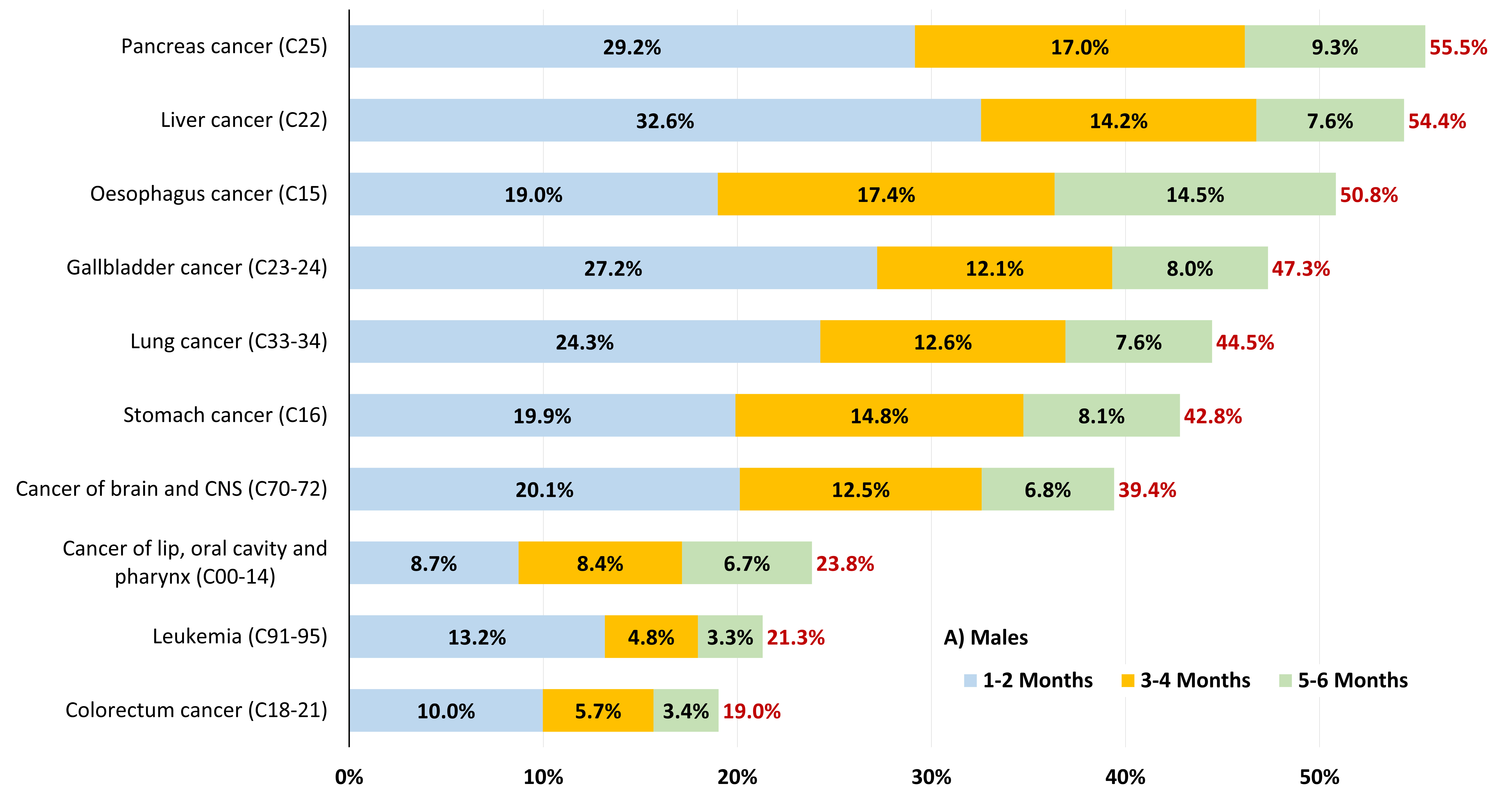

Supplement: Supplementary file 1 [file cancers-17-01670-s001.zip › Supplementary Figure S2A.png]

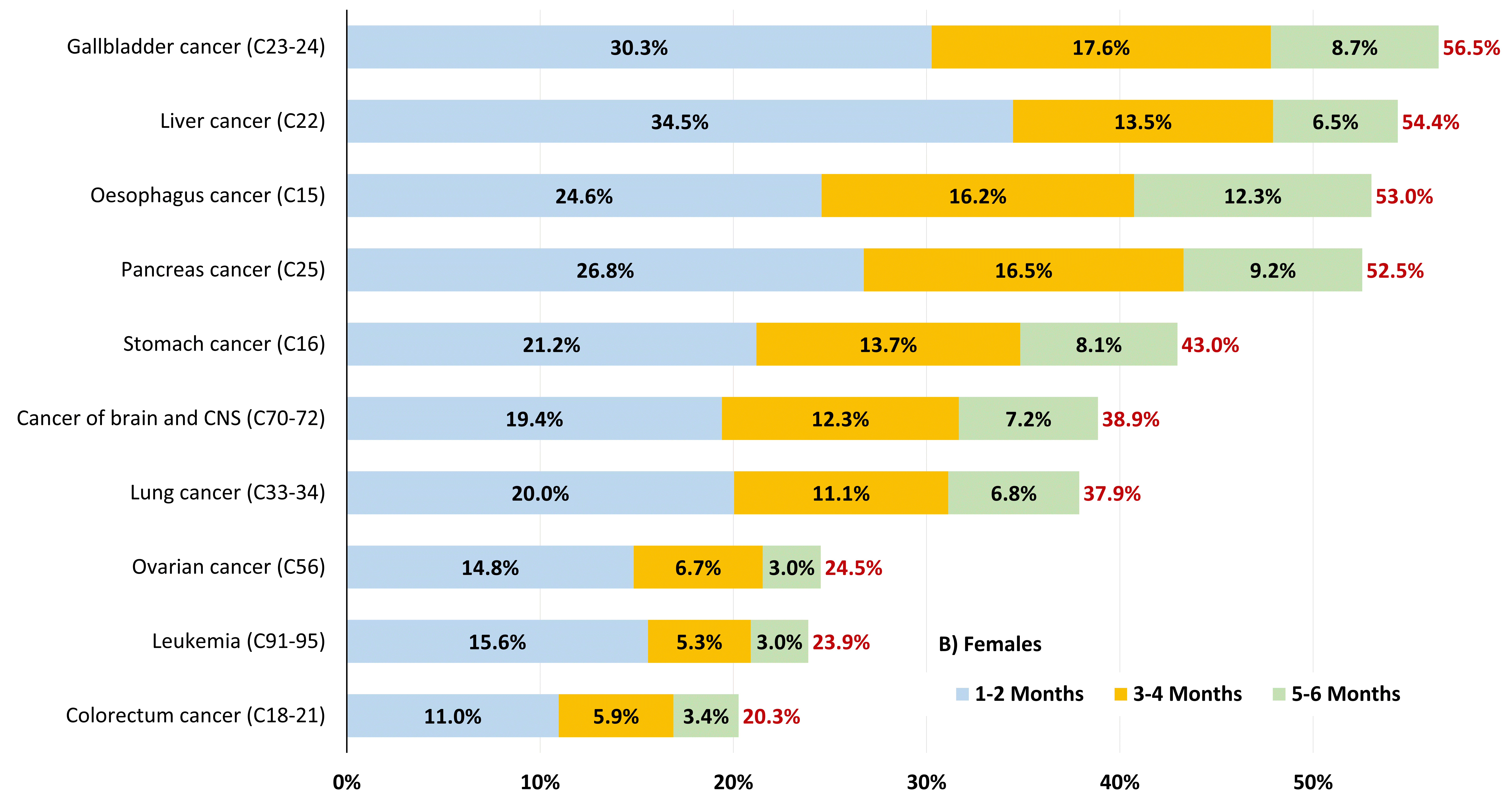

Supplement: Supplementary file 1 [file cancers-17-01670-s001.zip › Supplementary Figure S2B.png]

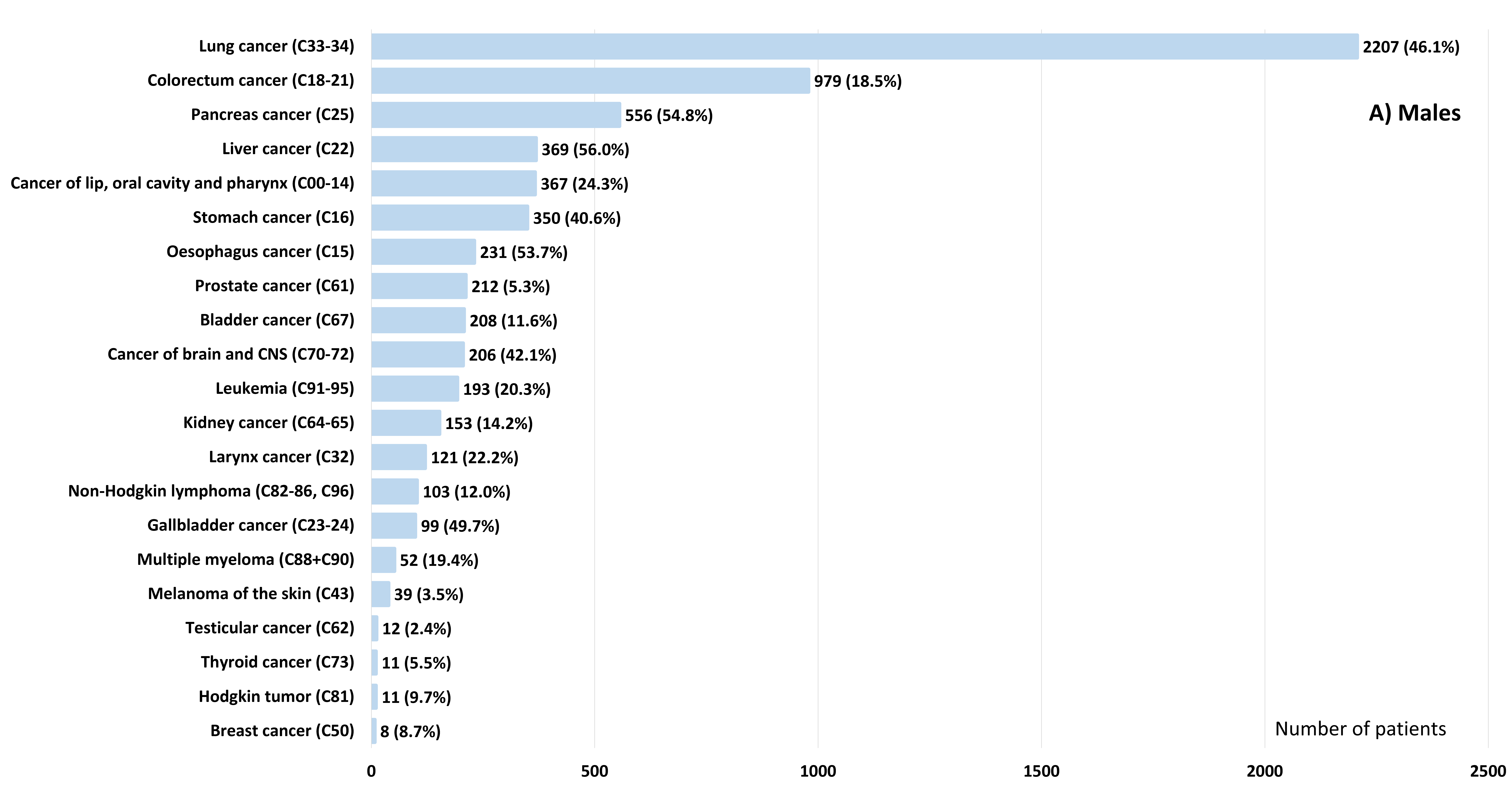

Supplement: Supplementary file 1 [file cancers-17-01670-s001.zip › Supplementary Figure S3A.png]

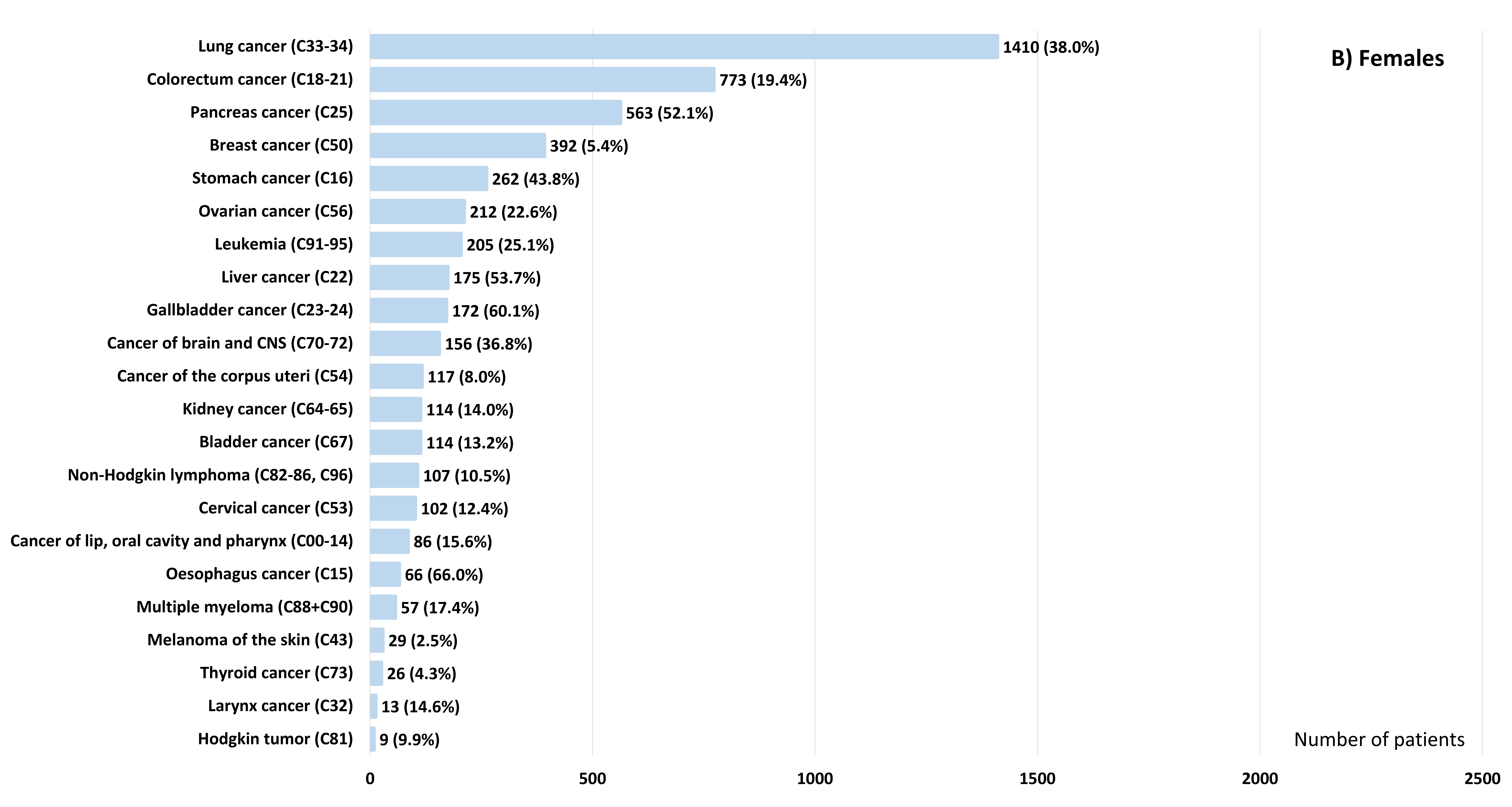

Supplement: Supplementary file 1 [file cancers-17-01670-s001.zip › Supplementary Figure S3B.png]
